# Comparing the accuracy of an ultrasound height measurement device with a wooden measurement board among children aged 2–5 years in rural Lao People’s Democratic Republic: A methods-comparison study

**DOI:** 10.1371/journal.pone.0289514

**Published:** 2023-11-17

**Authors:** Shan Huang, Joel Conkle, Caroline S. E. Homer, Sengchanh Kounnavong, Khampheng Phongluxa, Joshua P. Vogel

**Affiliations:** 1 Maternal, Child and Adolescent Health Program, Burnet Institute, Melbourne, Australia; 2 Department of Public Health and Preventative Medicine, Monash University, Melbourne, Australia; 3 Division of Data, Analytics, Planning and Monitoring, UNICEF, New York, New York, United States of America; 4 Lao Tropical and Public Health Institute, Ministry of Health, Vientiane, Lao PDR; Projahnmo Research Foundation, BANGLADESH

## Abstract

**Background:**

Height is a key component of nutrition assessments in children from limited-resource settings. This study aimed to assess whether handheld digital ultrasound devices for measuring children’s height provide comparable accuracy to traditional measurement boards, which are bulky and difficult to transport.

**Methods:**

We trained 12 health workers to measure the standing height of 222 children aged 2–5 years in rural Lao People’s Democratic Republic using both the ultrasound device and measurement board. The Bland-Altman method was used to depict limits of agreement and potential bias. We reported the technical error of measurement (TEM) for precision and accuracy, then assessed these results against the Standardized Monitoring and Assessment for Relief and Transition (SMART) Manual 2.0 and the WHO Multicentre Growth Reference Study (MGRS).

**Results:**

The average difference between the ultrasound and board measurements was 0.096 cm (95% limits-of-agreement: 0.041cm, 0.61cm) with a systematic bias of 0.1cm (95% confidence interval: 0.067cm, 0.134cm), suggesting the ultrasound measurements were slightly higher than those from the board. The ultrasound and board TEMs for precision were 0.157cm and 0.091cm respectively. The accuracy TEM was 0.205cm. All TEMs were within SMART and WHO MGRS limits.

**Conclusion:**

The ultrasound device is comparable to the measurement board among standing Lao children aged 2–5 years for precision and accuracy TEMs but showed a bias of 0.1cm. Further studies are required to assess whether calibration can minimise this bias and determine the ultrasound’s accuracy on recumbent length for infants and younger children.

## Introduction

Height and weight are basic anthropometric measurements that have long been used as indicators of childhood nutrition status [1–3]. These two common anthropometric measures are used to monitor a child’s growth and development, and to calculate subnational, national, and international estimates of undernutrition and overnutrition. Rates of stunting (low height-for-age) and wasting (low weight-for-height) in children under five years at a population level, are used by local and national governments to allocate resources for programs and activities to improve outcomes for childhood nutrition [4]. Estimation of these rates depends on accurate height and weight measurement [5].

While digital scales are routinely used to measure a child’s weight, measurement boards, or stadiometers are used to measure height, with readings done manually [6]. These boards are bulky, difficult to transport, costly, and prone to measurement errors in reading and recording reliable height measurements [7]. These errors can be attributed to the design of the boards, as well as incorrect use e.g. incorrect positioning of the child against the board, difficulty in seeing measurements etched onto the board, reading measurements at incorrect angles, and variability between different measurers [7]. Additionally, children aged under five years can be difficult to measure accurately as they tend to not stand or lie still while measurements are being taken [8].

Digital ultrasonic devices to measure length are used in the construction industry. Such devices are small, handheld, and potentially simpler to use compared to the bulky measurement board. However, their accuracy and precision for monitoring child growth has not been adequately reviewed or investigated. For ultrasound devices available on the market, we found no evidence regarding their use in children [9–11]. A few non-marketed devices have been formally tested in research settings with varying success [12–15]. Digital height measurement devices that are commercially available range from USD 25–50 per unit [9–11], making them significantly cheaper than measurement boards (at USD 114–259 each) [16]. If digital height tools were shown to be valid in clinical and survey settings, this could reduce the cost of child height surveillance programs. Using global UNICEF procurement figures from 2012–2016, potential cost savings could be up to USD 3–7 million annually [7].

This study aimed to investigate the feasibility of a digital ultrasound device called One Grows™, to measure height in children aged two to five years old. We hypothesised that this device would be easier to use, clearer to read and show acceptable accuracy and precision in a limited-resource context when compared to the measurement board.

## Materials and methods

### Study design and sample size calculation

We used a methods-comparison study design to assess the difference between the less-established digital ultrasound device One Grows™ against the current standard, the wooden measurement board prescribed by UNICEF; the latter is currently the primary tool used to measure height in low-resource contexts internationally [17]. In 2017, UNICEF released a Target Product Profile (TPP) with recommendations for novel height measurement devices [7]. One criterion for new products was an accuracy of 0.3cm. We used this level of precision as the maximum allowable variation between the ultrasound and the measurement board. Applying this clinical delta of ±0.3cm, assuming a zero bias, 80% power, 95% confidence interval and 10% attrition/missing data, we calculated a sample size of 220 children. Using the Bland-Alman statistical methods for assessing agreement between two methods of measurement [18], this sample size allowed us to provide statistical inference for an approximate maximum standard deviation of the difference in measurements of 0.127cm and a 95% limit-of-agreement between the two methods of 0.249cm.

### Ethical considerations

This study was approved by the Ethics Review Board of the Alfred Hospital in Melbourne, Australia (ID: 142/22), and the National Ethics Committee for Health Research from the Ministry of Health, Vientiane, Lao PDR (ID: 2022.15). No identifying data from the participants were collected. Prior to the study taking place, permission was sought by the local study team to conduct the study from the relevant district authorities and each study site. In the weeks leading up to the data collection taking place, authorities from the study sites informed the parent and caregivers of the participants of the purpose of our study. On the morning of the data collection, written informed consent was received from each child’s parent/caregiver at the study sites before measurements were taken.

### Study setting and participants

**Fig 1:**
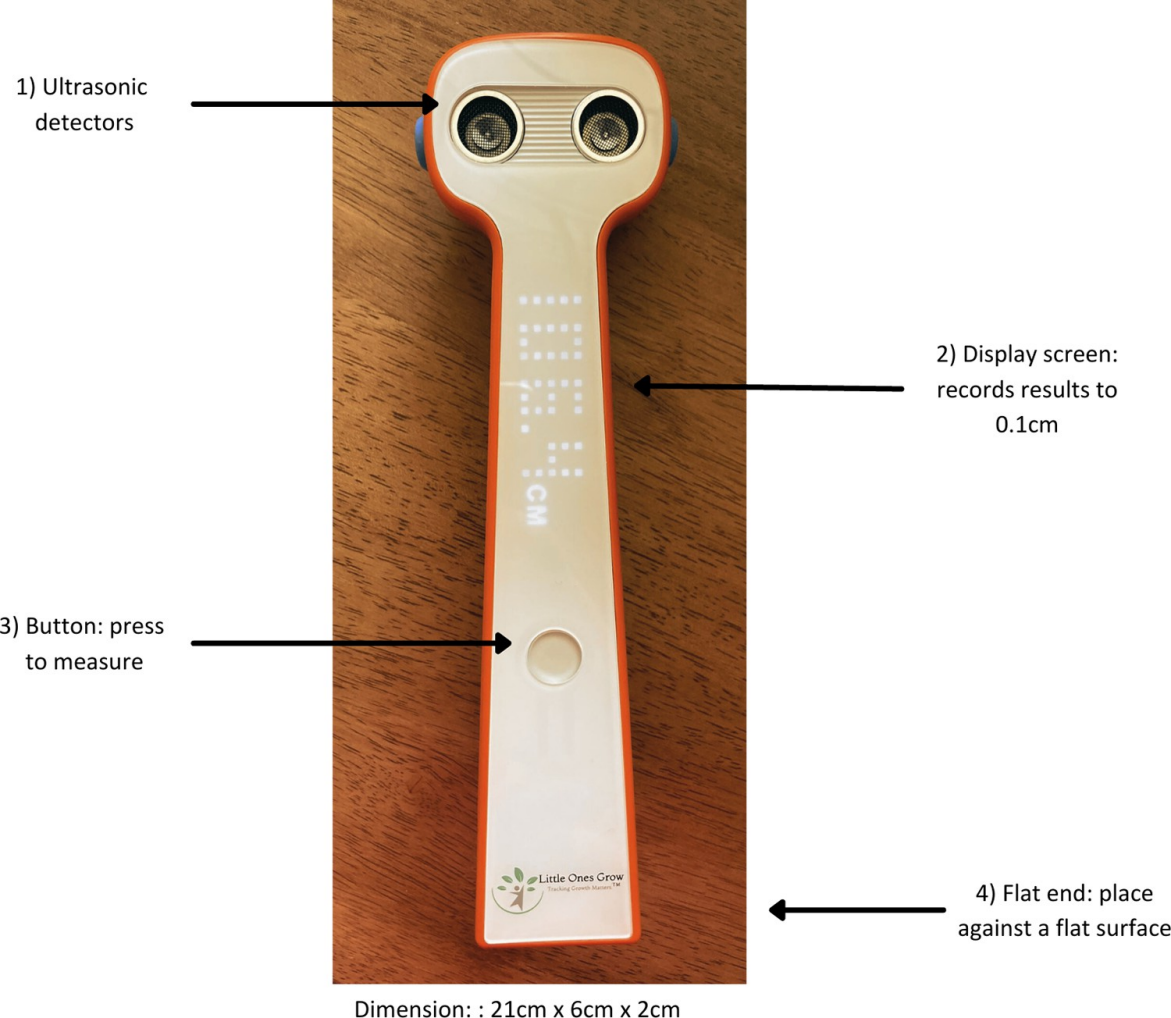
Photograph indicating aspects of the One Grows™ device. Aspects include 1) ultrasonic detectors, 2) display screen, 3) button for taking measurements, 4) flat bottom end to place against a flat surface. The dimension of the device is length: 21cm, width: 6cm and depth: 2cm.

We conducted this study in the district of Feuang in Vientiane Province, Lao People’s Democratic Republic (Lao PDR). Feuang is a district located 110 km by road from Vientiane capital city. According to the latest census from 2015, its population was just over 41,000 and 72% of the district was considered rural [19]. We recruited 222 children aged 2–5 years, who could stand independently without assistance. We compared a novel One Grows™ (INBCA Medical Corp, Shenzhen, China) ultrasound measurement device to the three-piece wooden height measurement board (UNICEF Supply Catalogue No: S0114540) [16], which is standard practice in Lao PDR. The One Grows™ is not currently patented (Fig 1) and measures height by sending ultrasound beams from the head of the device to a hard surface. To measure standing height, the device is placed perpendicularly against a wall, facing down on top of the child’s head. The child is then asked to step away from their position and the measurer presses the button on the device to allow it to take the measurement. The measurement is converted into centimetres (to 1 decimal place) by the device by calculating the time it takes for the beam to bounce to/from the hard surface. The comparator was a wooden measurement board—this is a standardised tool procured in Laos from UNICEF. It is the standard tool used in low-resource contexts to measure height and length in nutrition surveys and in clinical settings [20].

Both tools were used to measure children’s standing height only, not recumbent length. Participating children were identified and recruited from two kindergartens and three villages near the local health centre, deemed suitable study sites by the local authorities. Additionally, these kindergartens and local villages were chosen based on their proximity to the health centre and because health teams routinely perform community health outreach activities, including child height measurement at these locations [21].

### Training

We trained 12 health centre staff from the Feuang District and two study supervisors from the Lao Tropical and Public Health Institute (TPHI) on how to use the One Grows™ device. The Lao TPHI is the primary public health research body in the country. They are a branch of the Ministry of Health and have been operational in a research and teaching capacity since 1999. When conducting research, the local level government encourages the use of local health workers to build their capacity for research while being familiar with local contexts. In Lao PDR, health centre staff have three years of tertiary level education and are trained as ‘Medical Assistants’. They are responsible for local public health activities, including maternal and child health promotion and community-based nutritional screening. As such, all those trained had previous experience using the measurement board but no experience with the ultrasound device.

The Chief Investigator (a public health nutritionist and clinical dietitian) delivered a two-day training with training materials that included a training manual on the application and maintenance of One Grows™ and a simple instruction card for easy reference during data collection. Aspects such as correct head and feet positioning (the same when using the measurement board) were key aspects of the training and well-practiced using the One Grows™ device.

Given previous experience, no additional training on using the measurement board was provided to the team. However, a standardisation test on the use of the measurement board was administered to the health centre staff according to WHO protocols [1]. The senior measurer was a study supervisor from the Lao TPHI team who is an experienced anthropometrist. Based on standardisation protocols, if a lead measurer showed a precision Technical Error of Measurement (TEM) of ≥0.4cm (our senior measurer had a TEM of 0.6cm), the criterion for trainee anthropometrists passing the standardization exercise was based on precision TEM within the limits of the Standardized Monitoring and Assessment for Relief and Transition (SMART) Manual 2.0 [22]. Based on the standardization results, six of the twelve trained staff passed the protocol standards and became the measurers for data collection (precision TEMs between 0.6cm– 0.8cm). Since each team required a measurer and a scribe (someone to write down the result) the remaining six staff became measurement recorders. In total, 6 teams of two (one measurer, one scribe) were formed to collect data.

### Data collection

**Fig 2:**
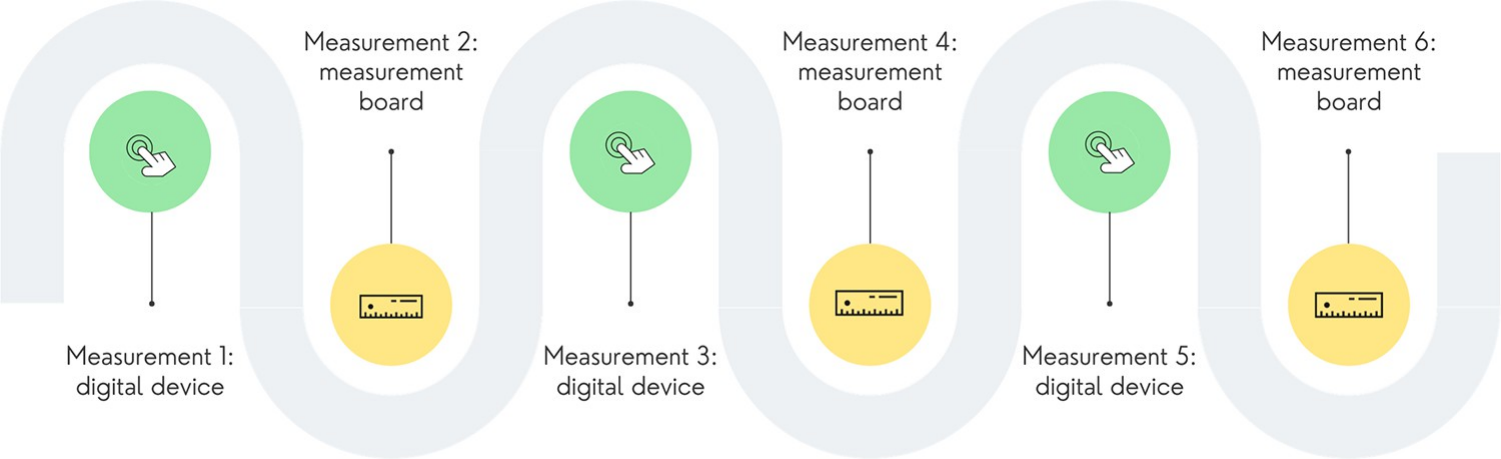
Measurement process for one child.

Data collection took place between 6–21 June 2022, immediately following the training. Written consent from the parent/caregiver was provided to the study team before any measurements were taken from the children. Children were selected in a random order to be measured. For each child, a total of six measurements were taken by a single enumerator–three measurements using the measurement board, and three using the One Grows™ device. The measurement process (Fig 2) alternated between the devices for each child to reduce the potential for recall bias should the measurer use the same device to measure the same child repeatedly one after another. The process then alternated in order again with the next child; for example, if child one was measured with the ultrasound device first, child two would be measured with the height board first and so on. Two study supervisors provided full-time supervision and ensured consistency in following the measurement process for each child. They also monitored data quality and ensured accurate recording of measurements.

### Statistical methods

All measurements collected were recorded manually on a paper data collection form (one form per child) and entered into a REDCap digital form by the study supervisors. REDCap is an electronic data capture tool, hosted and managed by Burnet Institute. It is a secure, web-based software platform designed to support data capture for research studies [23,24]. All data were downloaded from REDCap and analysed using StataSE 17 (StataCorp, College Station, TX, USA) software and Microsoft Excel.

We analysed our data based on the recommendations from the World Health Organization (WHO) Multicentre Growth Reference Study (MGRS) and the SMART Manual 2.0 [20,22]. The MGRS created the global WHO Growth Standards and guided how anthropometric measurements should be standardised. The data collectors and supervisors did not have these reference standards on hand during the data collection process. We followed the MGRS protocols for precision and accuracy analysis where we took the first two measurements by one measurer for both tools except where the first two measurements exceeded the maximum allowable difference of 0.7cm, the third measurement collected was used, and we analysed these measurements for precision and accuracy [20].

The precision and accuracy results were compared with the standards set by the SMART manual which represents the acceptable limits for the respective TEM [22]. These are shown in Table 1. Both the MRGS and SMART are widely accepted as standard for performing anthropometric measurements from children.

**Table 1:** SMART acceptable limits for TEMs of precision and accuracy.

Parameter for height (cm)	Good	Acceptable	Poor	Rejected
**Precision for TEM**	<0.4	<0.6	<1.2	≥1.2
**Accuracy TEM**	<0.4	<0.6	<1.4	≥1.4

TEMs are commonly used in anthropometric assessments as a measure of accuracy and precision, and they can detect levels of variation from repeated measurements of the same individual, by the same measurer (intra-rater measurements) [25]. Its’ interpretation is that the differences between repeated measurements will be within ± the value of TEM two-thirds (66%) of the time, and 95% of the differences will be within ±2 ×TEM [25]. The lower the TEM, the smaller the variation in the repeated measurements. Hence, the TEM assesses the spread of measurements taken for the individual child being measured.

For this study, precision and accuracy of measurements were analysed by calculating TEMs (as per $TEM=\sqrt{\frac{{\sum D}^{2}}{2N}}$ where D is the difference between the measurements taken and N is the total number of participants).

We define precision as the TEM between measurements taken with the same device to assess the consistency of the measurements taken. We define accuracy as the TEM between measurements taken with different devices, comparing the One Grows™ device to the measurement board [22].


$$TEM\;Precision=\sqrt{\frac{{\sum Difference\;between\;two\;measurements\;of\;the\;same\;device}^{2}}{2N}}$$

$$TEM\;Accuracy=\sqrt{\frac{{\sum Difference\;between\;two\;averages\;from\;different\;devices}^{2}}{2N}}$$

We used StataSE 17 for the Bland-Altman analysis and MS Excel to create the Bland-Altman plot. We compared the difference between the measurement devices across children of varying heights and ages, as well as the assessment of systematic bias. The Bland-Altman plot is a common way to represent results comparing two measurement methods [26]. The x-axis of the Bland-Altman plot is the average height measured by both the ultrasound device and the measurement board using the MGRS method, and the y-axis is the difference of the average ultrasound measurements subtracted from the average board measurements in centimetres. The mean difference between the average measurements for both devices was then calculated to assess systematic bias in order to assess how closely the ultrasound device is measuring to the measurement board. The upper and lower limits of agreement were plotted as two standard deviations from the mean difference of the two devices. We used Pitman’s Test of difference in variance to determine if the difference between methods (y-axis) changed at different levels of average height (x-axis), where average height can be used as a proxy for age groups.

## Results

### Characteristics of study participants

In total, 222 children aged between 24–60 months (mean age 41.3 months**± 11.0 months**) participated. Of the 222 children, 52.7% (n = 117) were male and 47.3% (n = 105) were female. 20.3% of children were aged between 24–35 months (n = 45), 39.6% aged between 36–47 months (n = 88), 26.6% aged between 48–59 months (n = 59) and 13.5% aged at 60 months (n = 30) (Table 2).

**Table 2:** Sample characteristics.

**Age in months, mean ± standard deviation**	**41.3 ± 11.0**
**Age group, no (%)**	
**24–35**	**45 (20.3)**
**36–47**	**88 (39.6)**
**48–59**	**59 (26.6)**
**60**	**30 (13.5)**
**Sex, no (%)**	
**Female**	**105 (47.3)**

### Comparison of measurement methods using the Bland-Altman method

The summary statistics are presented in Tables 3 and 4. The average of all three measurements taken using the digital ultrasound device was 97.04cm (range, 77.63 to 119.33cm; SD = 8.22cm) compared to the average of all three measurements taken with the measurement board of 96.94cm (range, 77.03 to 119.27cm; SD = 8.21cm).

**Table 3:** Summary statistics for all measurements taken using both devices (n = 222 children).

	Mean	Standard Deviation	Min Measurement	Max Measurement	Standard Error
**Ultrasound measurements**					
**Ultrasound Measurement 1 (cm)**	97.06	8.22	77.90	119.30	0.55
**Ultrasound Measurement 2 (cm)**	97.04	8.21	77.60	119.40	0.55
**Ultrasound Measurement 3 (cm)**	97.03	8.23	77.40	119.30	0.55
**Average of all Ultrasound Measurements (cm)**	97.04	8.22	77.63	119.33	0.55
**Board measurements**					
**Board Measurement 1 (cm)**	96.96	8.21	77.00	119.20	0.55
**Board Measurement 2 (cm)**	96.93	8.22	77.00	119.30	0.55
**Board Measurement 3 (cm)**	96.93	8.21	77.10	119.30	0.55
**Average of all Board Measurements (cm)**	96.94	8.21	77.03	119.27	0.55

**Table 4:** Difference of measurements between ultrasound and board measurements (cm).

	Mean difference	Difference range	Standard Deviation of difference	Limits-of-agreement
**Difference of measurements**	0.1	-0.60 and 1.05	0.28	-0.46, 0.65

**Fig 3:**
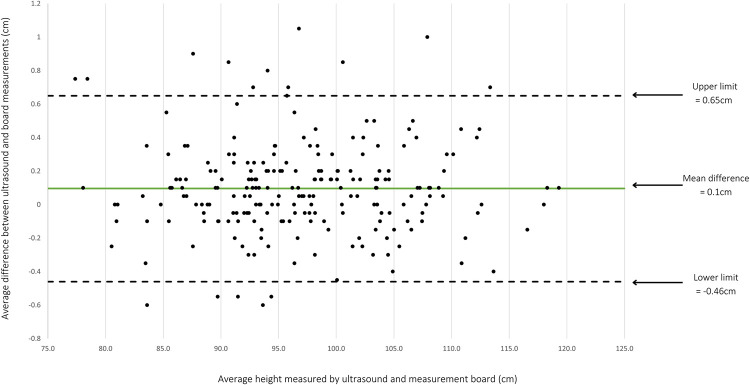
Bland-Altman plot. The average height measurements of the ultrasound readings were subtracted from the average of measurement board readings for each child (y-axis) plotted against the average height measured for each child by both tools (x-axis).

The Bland-Altman plot in Fig 3 indicates that the 95% limits-of-agreement between the board and the ultrasound device using the MRGS method was between -0.46cm to 0.65cm (SD = 0.28cm). There was a range of difference when we subtracted the ultrasound measurements from the board measurements, between -0.6 and 1.05cm. The Bland-Altman analysis using StataSE 17 calculated an overall mean difference, or systematic bias of 0.1cm (95%CI 0.06 to 0.13cm), indicating that the ultrasound device was consistently measuring the children 0.1cm taller than the board in this population.

Pitman’s test results show that the difference between the board and the ultrasound device did not significantly differ by average height (r = 0.002, p = 0.973). This indicates that a child’s age had no relationship with the difference in height measurements between the two devices.

### Precision and accuracy

#### Precision

As shown in Table 5, the precision TEM for the ultrasound device was 0.157cm (females: 0.157cm, males: 0.158cm), and the precision TEM for the height board was 0.091cm (females: 0.101cm, males: 0.080cm). Based on the standards set by the SMART manual, TEMs for both the ultrasound device and height board were <0.4cm, indicating ‘good’ precision.

**Table 5:** Precision and accuracy of ultrasound and measurement board.

	Females (n = 105)	Males (n = 117)	All (n = 222)
**Precision TEM Digital Ultrasound**	0.157cm	0.158cm	0.157cm
**Precision TEM Measurement Board**	0.101cm	0.080cm	0.091cm
**Accuracy TEM between Digital and Measurement Board**	0.227cm	0.183cm	0.205cm

#### Accuracy

The accuracy between the ultrasound and the height board was 0.205cm (females: 0.227cm, males: 0.183cm). Similarly, based on the SMART standards, these accuracy levels also indicated ‘good’ accuracy between the two devices, with girls showing a slightly greater accuracy than boys.

#### Team results

Overall, the teams performed better using measurement boards (precision TEM: 0.06cm– 0.12cm) than the ultrasound (precision TEM: 0.11cm– 0.26cm). The accuracy range was 0.14cm– 0.26cm. The sample size between teams varied greatly (between 19–75 children) as our study was not designed to assess TEM among the teams, but these results do indicate that both tools were reliable and within acceptable limits.

## Discussion

This study of 222 children in Lao PDR compared measurements taken using the One Grows™ ultrasound device and the UNICEF wooden measurement board (standard of care). For reliability, the TEM results were within acceptable SMART and WHO MGRS limits, indicating that each device produces consistent results. The measurement board showed a slightly higher precision than the ultrasound device (TEM 0.091cm vs 0.157cm).

For accuracy, the TEM of 0.205cm was well within the acceptable limits of the SMART and WHO MGRS guidelines [20]. There was a small difference in bias TEM among girls and boys—the accuracy TEM among girls was higher by 0.044cm. Upon closer inspection of the measurements recorded using the ultrasound device, there were three occasions where the third measurement had to be used because the maximum allowable difference was exceeded among the girls, but only one occasion of this occurred when measuring the boys. None of the board measurements required adjusting (using a third measurement). During the study, we observed that girls often had long hair tied up in elaborate styles. It is possible that when the measurer releases a child’s hair to perform the measurement, their hair is still messy and might cause additional movement (and hence additional differences between measurements) when using the handheld ultrasound device. Conversely, the headpiece of the measurement board is heavy and thus may be steadier. Further investigation of possible sources of measurement-to-measurement variation when using a handheld ultrasound device is required.

The ultrasound device produced a systematic bias of 0.1cm and was shown to measure higher than the measurement board. However, it is noted that while traditional measurement boards are widely used, this is due to the lack of availability of other tools rather than measurement boards necessarily being a consistently superior standard. Without proper calibration of either the ultrasound device or the board, it is therefore difficult to determine whether the ultrasound device is measuring the child taller or the board is measuring the child shorter, and further studies would need to consider calibration of both devices.

When child height is measured in a clinical setting, usually only one measurement is taken. While the board showed slightly higher precision, our findings suggest the ultrasound device could be a more suitable choice for clinical use, particularly in low-resource settings, given its greater portability, lightweight nature, lower cost, and ease-of-use.

The 2017 UNICEF TPP [7] suggested a novel height measurement device needed to meet two essential requirements: firstly, to improve upon currently available measurement boards with a digital output; and secondly to use innovative technologies (such as ultrasound, infrared or laser). The One Grows™ device meets both essential UNICEF TPP essential requirements. It also meets several optional requirements including an associated mobile application (app) whereby the measurement taken by the device is automatically entered via Bluetooth technology, and requirements for operational conditions, portability, maintenance, physical characteristics, and affordability [7]. The app also allows for multiple measurements on multiple children. Our device also met the accuracy range set by the TPP, which was within 0.3cm for TEM. Our study showed that the One Grows™ device meets the main requirements from the UNICEF TPP except for a low battery indicator and assessments for commercialization requirements.

To our knowledge, this is the first study to compare this handheld ultrasound device with the measurement board. Few other ultrasound devices have been studied in detail on children, and none of these studies were done in the context of rural health workers in a low or middle-income country. In 1998, Watt, Pickering and Wales published a study with 18 children (ages unspecified) in the United Kingdom using the Gulliver G-100 ultrasound device, which showed an average bias between 0.74–0.88cm when compared with a Harpenden stadiometer [12]. In 1999, Glock et al also used the G-100 device to assess the growth hormone treatment of 101 children with severe dwarfism in Germany, with results showing an average bias of 0.49cm when compared to the Harpenden stadiometer [14]. In both studies, the difference in measurement between the devices was more than five times greater than in our study (0.1cm vs. >0.49cm). In 2013, Syafiq and Fikawati [15] tested the feasibility of a prototype measurement board using an ultrasound attachment (the P2B2D) on 53 infants in Malaysia compared to a plastic length board. While their inter-method TEM of 3.66cm is rejected based on SMART standards [22], it is noted that the study measured the length of infants rather than standing height as in or study. Length is known to be more difficult to measure correctly than standing height, as it requires careful positioning to ensure the child is appropriately stretched before taking the measurement [3]. In 2020, Cho et al. validated a handheld ultrasound device (InLab S50) similar to the One Grows™ device among 100 adults in South Korea, and reported similar findings [13]. When compared to the stadiometer, the InLab S50 had a mean bias of -0.15cm (95% limits-of-agreement: -1.69cm, 1.38cm), whereas in our study, the mean bias was 0.1cm (95% limits-of-agreement: -0.46cm, 0.66cm). By comparison, the InLab S50 consistently measured lower height compared to the board, while the One Grows™ measured greater than the board. Our study also showed smaller margins of difference compared to the InLab S50 device.

### Strengths and limitations

Our study demonstrated the One Grows™ ultrasound device performs valid height measurements in standing children 2–5 years of age, and that the measurements were as accurate and precise as wooden measurement boards. This is significant because previous studies evaluating ultrasound-based height measurement devices have not shown the same level of accuracy and preciseness. Strengths of this study included a large sample size (222 children), adherence to the WHO MGRS methodology, and the comparison of results to the SMART standards. This study is a novel approach to evaluating the use of ultrasound devices for measuring children’s height in low resource settings, performed by healthcare personnel with limited formal training who routinely perform child nutritional surveillance activities. The study setting and training approach closely reflect the real-world needs for novel, digital devices, and provide a way forward in investigating how handheld height measurement devices can be used to measure child stature in low resource contexts. The results demonstrate that this device may be an appropriate substitute for traditional measurement boards, highlighting an opportunity to research its feasibility in other contexts.

While our study findings demonstrate that the two devices were comparable to each other, a limitation was that we only measured children who could stand at 2–5 years of age–we did not test it on children under two years where recumbent length is measured. We also note that the comparison TEMs are set against children aged 0–5 years, not children aged 2–5 years. For recumbent length, this ultrasound device would measure length from foot to head, rather than height from head to ground. This would require further investigation to determine if the accuracy is similar in recumbent measurement. Our measurement process of alternating devices for the same child could contribute to measurers being biased by previous measurements. To reduce this bias, in future studies, it may be worth considering the measurement of an entire group of children once with both devices, then re-measuring the same group of children another time over. Our study design also did not permit assessment of inter-rater reliability as each child was only measured by one measurer so only intra-rater reliability (precision) was obtained. For inter-rater reliability to be assessed adequately, each child would require measurement from two or more measurers. While we did not formally assess the cost-effectiveness of the ultrasound device, it is cheaper than the measurement board (USD 50 compared to USD 114–259 from the UNICEF Supply Catalogue) [7]. We consider that a formal cost-effective evaluation that considers durability and recurrent costs would be useful to guide procurement decision-making.

## Conclusion

In a methods-comparison study of 222 children between 2–5 years old in Lao PDR, the One Grows™ ultrasound device showed good levels of precision and accuracy in measuring the height of standing children. However, there was a systematic bias between the devices, with the ultrasound measurement being 0.1cm greater than that of the measurement board. Future studies showing calibration of both devices should ascertain which of these devices measures the closest to the true height/length of a child; and whether the ultrasound device requires further recalibration. Particularly in limited-resource settings, this ultrasound device, which is handheld, lightweight, and comparably low-cost, could be a suitable alternative to measurements boards which are bulky and heavy to carry. Additional studies involving measurement of recumbent height in younger children are required, as well as further assessments to explore inter-rater reliability, and device performance in other settings.
