# Supplementary material for: Comparing the accuracy of an ultrasound height measurement device with a wooden measurement board among children aged 2–5 years in rural Lao People’s Democratic Republic: A methods-comparison study
Source: PLoS One. 2023 Nov 17;18(11):e0289514. doi: 10.1371/journal.pone.0289514 (PMC10656007; doi:10.1371/journal.pone.0289514)
Supplement: Peer review history

# Peer review: Decision Letter 0

**DOI:** 10.1371/journal.pone.0289514.r001

**Authors:** Sayedur Rahman

7 Sep 2023

PONE-D-23-15762Comparing the accuracy of an ultrasound height measurement device with a wooden measurement board among children aged 2-5 years in rural Lao People’s Democratic Republic: a methods-comparison study.PLOS ONE

Dear Dr. Huang,

Thank you for submitting your manuscript to PLOS ONE. After careful consideration, we feel that it has merit but does not fully meet PLOS ONE’s publication criteria as it currently stands. Therefore, we invite you to submit a revised version of the manuscript that addresses the points raised during the review process.

Please submit your revised manuscript by Oct 22 2023 11:59PM. If you will need more time than this to complete your revisions, please reply to this message or contact the journal office at plosone@plos.org. When you're ready to submit your revision, log on to https://www.editorialmanager.com/pone/ and select the 'Submissions Needing Revision' folder to locate your manuscript file.

Please include the following items when submitting your revised manuscript:

- A rebuttal letter that responds to each point raised by the academic editor and reviewer(s). You should upload this letter as a separate file labeled 'Response to Reviewers'.
- A marked-up copy of your manuscript that highlights changes made to the original version. You should upload this as a separate file labeled 'Revised Manuscript with Track Changes'.
- An unmarked version of your revised paper without tracked changes. You should upload this as a separate file labeled 'Manuscript'.

If you would like to make changes to your financial disclosure, please include your updated statement in your cover letter. Guidelines for resubmitting your figure files are available below the reviewer comments at the end of this letter.

If applicable, we recommend that you deposit your laboratory protocols in protocols.io to enhance the reproducibility of your results. Protocols.io assigns your protocol its own identifier (DOI) so that it can be cited independently in the future. For instructions see: https://journals.plos.org/plosone/s/submission-guidelines#loc-laboratory-protocols. Additionally, PLOS ONE offers an option for publishing peer-reviewed Lab Protocol articles, which describe protocols hosted on protocols.io. Read more information on sharing protocols at https://plos.org/protocols?utm_medium=editorial-email&utm_source=authorletters&utm_campaign=protocols.

We look forward to receiving your revised manuscript.

Kind regards,

Sayedur Rahman, MBBS, MMSc

Academic Editor

PLOS ONE

Journal Requirements:

When submitting your revision, we need you to address these additional requirements.

1. Please ensure that your manuscript meets PLOS ONE's style requirements, including those for file naming. The PLOS ONE style templates can be found at 

https://journals.plos.org/plosone/s/file?id=wjVg/PLOSOne_formatting_sample_main_body.pdf and 

https://journals.plos.org/plosone/s/file?id=ba62/PLOSOne_formatting_sample_title_authors_affiliations.pdf

2. We note that the grant information you provided in the ‘Funding Information’ and ‘Financial Disclosure’ sections do not match. 

When you resubmit, please ensure that you provide the correct grant numbers for the awards you received for your study in the ‘Funding Information’ section.

3. Thank you for stating the following in the Acknowledgments Section of your manuscript: 

"..The authors also acknowledges funding from the Burnet Institute to support this study."

Funding information should not appear in the Acknowledgments section or other areas of your manuscript. We will only publish funding information present in the Funding Statement section of the online submission form. 

Please remove any funding-related text from the manuscript and let us know how you would like to update your Funding Statement. Currently, your Funding Statement reads as follows: 

"This study was financially supported by the Burnet Institute and was awarded to Shan Huang. The funders had no role in the study design, data collection and analysis, decision to public, or preparation of hte manuscript."

Please include your amended statements within your cover letter; we will change the online submission form on your behalf.

4. Thank you for stating the following financial disclosure: 

"This study was financially supported by the Burnet Institute and was awarded to Shan Huang. The funders had no role in the study design, data collection and analysis, decision to public, or preparation of hte manuscript."

We note that one or more of the authors is affiliated with the funding organization, indicating the funder may have had some role in the design, data collection, analysis or preparation of your manuscript for publication; in other words, the funder played an indirect role through the participation of the co-authors. If the funding organization did not play a role in the study design, data collection and analysis, decision to publish, or preparation of the manuscript and only provided financial support in the form of authors' salaries and/or research materials, please do the following:

(1) Review your statements relating to the author contributions, and ensure you have specifically and accurately indicated the role(s) that these authors had in your study. These amendments should be made in the online form.

(2) Confirm in your cover letter that you agree with the following statement, and we will change the online submission form on your behalf: 

“The funder provided support in the form of salaries for authors [insert relevant initials], but did not have any additional role in the study design, data collection and analysis, decision to publish, or preparation of the manuscript. The specific roles of these authors are articulated in the ‘author contributions’ section.”

5. Please ensure that you refer to Figure 1 in your text as, if accepted, production will need this reference to link the reader to the figure.

6. Please include a copy of Table 2 which you refer to in your text on page 9.

**Additional Editor Comments:**

Please address the points raised by the reviewers in your revised manuscript. In the revised version, we strongly recommend that you provide sufficient information about the novel height measurement device (One Grows™) in the methods section which may include name of manufacturer, how the device works, patents, reference link, etc. You may consider adding a picture of the device to the manuscript. Please provide some information about the wooden measurement board too (i.e., locally made? standardized? any previous research that had used this measurement board? if yes, please cite pertinent articles). Table 2 (characteristics of study participants) is missing in the manuscript, please add the table. If you have collected sociodemographic information about the participants, please also include them in Table 2.

There are a few other issues that you need to address: 1) There are no intervention and control groups among the participants. Therefore, use of these terms (lines 81, 82) can be confusing to the readers. Please rephrase this sentence; 2) You have mentioned in line 88 that ‘Children were identified using convenience sampling’. However, you have described the sample size calculation in lines 95, 96, and also have stated that ‘Children were selected in a random order to be measured’ (line 132). Texts related to selection of the samples needs to be clearer and consistent throughout the paper; 3) Regarding standardization test of the data collectors, please mention who was the gold standard measurer. Were there inter- and intra-rater reliability tests done during the training session? If yes, please add a sentence about the findings; 4) Please correct the name of the statistical method in line 207, it should be Bland-Altman; 5) It might be better to split the section ‘Study design and ethics (line 90)’ into two distinct sections – ‘Study design and sample size calculation’ and ‘Ethical considerations’; 6) Study design has not been mentioned in the ‘Study design’ section, please clearly mention the design of the study; 7) In the ‘Study setting and participants’ section, please consider adding a few demographics of the Vientiane Province that may include geographical location, population, etc. 8) It would be good to provide information about the research group/institution/organization that conducted the study; 9) The subheading “Training on use of One Grows™ device and Standardization Exercise for measurement” can be shortened. It can be simply “Training”; 10) please consider using appropriate citations for the REDCap and StataSE 17 (line 147). Further, we would like to request that you revise the text to fix the grammatical errors and improve the language usage and overall readability of the manuscript.

[Note: HTML markup is below. Please do not edit.]

Reviewers' comments:

Reviewer's Responses to Questions

**Comments to the Author**

1. Is the manuscript technically sound, and do the data support the conclusions?

The manuscript must describe a technically sound piece of scientific research with data that supports the conclusions. Experiments must have been conducted rigorously, with appropriate controls, replication, and sample sizes. The conclusions must be drawn appropriately based on the data presented. 

Reviewer #1: Partly

Reviewer #2: Partly

**********

2. Has the statistical analysis been performed appropriately and rigorously? 

Reviewer #1: Yes

Reviewer #2: Yes

**********

3. Have the authors made all data underlying the findings in their manuscript fully available?

The PLOS Data policy requires authors to make all data underlying the findings described in their manuscript fully available without restriction, with rare exception (please refer to the Data Availability Statement in the manuscript PDF file). The data should be provided as part of the manuscript or its supporting information, or deposited to a public repository. For example, in addition to summary statistics, the data points behind means, medians and variance measures should be available. If there are restrictions on publicly sharing data—e.g. participant privacy or use of data from a third party—those must be specified.

Reviewer #1: Yes

Reviewer #2: No

**********

4. Is the manuscript presented in an intelligible fashion and written in standard English?

PLOS ONE does not copyedit accepted manuscripts, so the language in submitted articles must be clear, correct, and unambiguous. Any typographical or grammatical errors should be corrected at revision, so please note any specific errors here.

Reviewer #1: Yes

Reviewer #2: Yes

**********

5. Review Comments to the Author

Please use the space provided to explain your answers to the questions above. You may also include additional comments for the author, including concerns about dual publication, research ethics, or publication ethics. (Please upload your review as an attachment if it exceeds 20,000 characters)

Reviewer #1: This paper compares two methods, a wooden height meter board and an ultrasound device, by measuring height in children aged 2-5 years in Lao PDR.

The article describes the results, assessing TEM for precision and accuracy and comparing the results with the WHO MGRS and SMART manuals.

The focus of the paper is well phrased.

I recommend that this paper be accepted after minor revision.

Minor comments:

1. It is mentioned that 12 health workers were trained in the measurement of height using ultrasound equipment (line 22). On the other hand, it is noted that only 6 of these workers passed the protocol standards and served as measurers in the study (line 125). Therefore, the above statement may be misleading.

2. Ethical aspects need to be described in detail. It is stated that it was convenience sampling (line 88) and that consent was obtained from parents or caregivers (line 89), but the specifics are not clear from these descriptions. Although this study involves recruiting subjects in kindergartens and villages in Lao PDR, it is unclear whether sufficient consideration was given to the subjects regarding their permission to participate. The fact that the health team routinely conducts outreach activities may have influenced subjects' willingness to participate. Therefore, the measures to address this should also be described.

3. The text does not describe any differences among assessors. It is anticipated that this data would provide a better understanding of the comparison between the two height measurement methods.

4. The use of ultrasound device in resource-limited settings is described (line 272). However, durability as well as convenience are important factors in making better choices for clinical use in these environments. Is this point included in the discussion?

Reviewer #2: There are several points in the manuscript to be made clearer.

1. Although the authors desribe "Aspects such as correct head and feet positioning (the same when using the measurement board) were key aspects" in lines 116-117, the height measurement by One Grows may be not familiar to readers. Please, add the further imformation on the logic of One Grows.

2. In line 126, the authors describe "six measurement teams". Were there differences in precision and accuracy among the teams? Please, mension them in Results.

3. In this study, one team measured height with the two methods alternatively but consequtively. The staff could memorize the measured height, resulting in the reduced difference among six measurement. Please, discuss the effect.

4. The authors stated that the subjects were 222 children in the text, but Table 5 shows 210 females, 134 males, and 444 in total.

5. In line 261, the authors stated "The ultrasound device produced a systematic bias of 0.1cm", as well as in line 30, but there is no description on it in Results.

Technical errors are as below.

1. In line 32, "The ultrasound and board TEMs for precision were 0.157cm and 0.113 respectively. ", "cm" may be needed after "0.113".

2. There is no Table 2 after line 201.

3. It is better to replace "Table 5: Precision and bias of ultrasound and measurement board" with "Table 5: Precision and accuracy of ultrasound and measurement board".

**********

6. PLOS authors have the option to publish the peer review history of their article (what does this mean?). If published, this will include your full peer review and any attached files.

If you choose “no”, your identity will remain anonymous but your review may still be made public.

**Do you want your identity to be public for this peer review?** For information about this choice, including consent withdrawal, please see our Privacy Policy.

Reviewer #1: **Yes: **Yasunori Ichimura

Reviewer #2: **Yes: **Nobuyuki Hamajima

**********

[NOTE: If reviewer comments were submitted as an attachment file, they will be attached to this email and accessible via the submission site. Please log into your account, locate the manuscript record, and check for the action link "View Attachments". If this link does not appear, there are no attachment files.]

While revising your submission, please upload your figure files to the Preflight Analysis and Conversion Engine (PACE) digital diagnostic tool, https://pacev2.apexcovantage.com/. PACE helps ensure that figures meet PLOS requirements. To use PACE, you must first register as a user. Registration is free. Then, login and navigate to the UPLOAD tab, where you will find detailed instructions on how to use the tool. If you encounter any issues or have any questions when using PACE, please email PLOS at figures@plos.org. Please note that Supporting Information files do not need this step.

# Author comment: Author response to Decision Letter 0

**DOI:** 10.1371/journal.pone.0289514.r002

10 Oct 2023

1. Please ensure that your manuscript meets PLOS ONE's style requirements, including those for file naming. The PLOS ONE style templates can be found at 

https://journals.plos.org/plosone/s/file?id=wjVg/PLOSOne_formatting_sample_main_body.pdf and 

https://journals.plos.org/plosone/s/file?id=ba62/PLOSOne_formatting_sample_title_authors_affiliations.pdf

Response: Thank you for this guidance. Apologies that I had missed some of the formatting guidelines. I have corrected these as per your instructions. Please note that all page and line references in our responses and revisions herewith are based on the UNMARKED copy of the manuscript.

2. We note that the grant information you provided in the ‘Funding Information’ and ‘Financial Disclosure’ sections do not match. 

When you resubmit, please ensure that you provide the correct grant numbers for the awards you received for your study in the ‘Funding Information’ section.

Response: The funds for this project were awarded through a small project grant received from a competitive, peer-reviewed process within Burnet Institute. There are no grant numbers associated with funding from within the institute but I have made it clear it was received in 2022. 

3. Thank you for stating the following in the Acknowledgments Section of your manuscript: 

"..The authors also acknowledges funding from the Burnet Institute to support this study."

Funding information should not appear in the Acknowledgments section or other areas of your manuscript. We will only publish funding information present in the Funding Statement section of the online submission form. 

Please remove any funding-related text from the manuscript and let us know how you would like to update your Funding Statement. Currently, your Funding Statement reads as follows: 

"This study was financially supported by the Burnet Institute and was awarded to Shan Huang. The funders had no role in the study design, data collection and analysis, decision to public, or preparation of the manuscript."

Please include your amended statements within your cover letter; we will change the online submission form on your behalf.

Response: Thank you for this guidance. This statement has been removed from the manuscript and I have included the amended statement in my cover letter.

4. Thank you for stating the following financial disclosure: 

"This study was financially supported by the Burnet Institute and was awarded to Shan Huang. The funders had no role in the study design, data collection and analysis, decision to public, or preparation of the manuscript."

We note that one or more of the authors is affiliated with the funding organization, indicating the funder may have had some role in the design, data collection, analysis, or preparation of your manuscript for publication; in other words, the funder played an indirect role through the participation of the co-authors. If the funding organization did not play a role in the study design, data collection and analysis, decision to publish, or preparation of the manuscript and only provided financial support in the form of authors' salaries and/or research materials, please do the following:

(1) Review your statements relating to the author contributions and ensure you have specifically and accurately indicated the role(s) that these authors had in your study. These amendments should be made in the online form.

(2) Confirm in your cover letter that you agree with the following statement, and we will change the online submission form on your behalf: 

“The funder provided support in the form of salaries for authors [SH] but did not have any additional role in the study design, data collection and analysis, decision to publish, or preparation of the manuscript. The specific roles of these authors are articulated in the ‘author contributions’ section.”

Response: The funds for this project were awarded through a small project grant received from a competitive, peer-reviewed process within Burnet Institute. I have updated this in the online submission as well as the Cover Letter. 

5. Please ensure that you refer to Figure 1 in your text as, if accepted, production will need this reference to link the reader to the figure.

Response: Thank for noting this. This has been changed and Figures 1 and 2 are now corrected cited in the manuscript and the attachments.

6. Please include a copy of Table 2 which you refer to in your text on page 9.

Response: Thank you for noting this. The Table 2 is now included (page 11).

Additional Editor Comments:

Please address the points raised by the reviewers in your revised manuscript. In the revised version, we strongly recommend that you provide sufficient information about the novel height measurement device (One Grows™) in the methods section which may include name of manufacturer, how the device works, patents, reference link, etc. You may consider adding a picture of the device to the manuscript. Please provide some information about the wooden measurement board too (i.e., locally made? standardized? any previous research that had used this measurement board? if yes, please cite pertinent articles). Table 2 (characteristics of study participants) is missing in the manuscript, please add the table. If you have collected sociodemographic information about the participants, please also include them in Table 2.

Response: Thank you for your suggestions. We have included further information on the One Grows device as well as the measurement board. We have also included an additional Figure which is a photo of the One Grows device (page 6). Table 2 has also been included as per response above (page 11). 

There are a few other issues that you need to address: 

1) There are no intervention and control groups among the participants. Therefore, use of these terms (lines 81, 82) can be confusing to the readers. Please rephrase this sentence; 

Response: This sentence has been rephrased to ‘We compared a novel One Grows™ (INBCA Medical Corp, Shenzhen, China) ultrasound measurement device to the three-piece wooden height measurement board (UNICEF Supply Catalogue No: S0114540) (16), which is the standard practice in Lao PDR.’ (Page 5, line 107-109)

2) You have mentioned in line 88 that ‘Children were identified using convenience sampling’. However, you have described the sample size calculation in lines 95, 96, and also have stated that ‘Children were selected in a random order to be measured’ (line 132). Texts related to selection of the samples needs to be clearer and consistent throughout the paper; 

Response: Thank you for this comment – we agree this should be clarified. I have deleted the sentence ‘Children were identified using convenience sampling’ and rephrased the original text to ‘Participating children were identified and recruited from two kindergartens and three villages near the local health centre, deemed suitable study sites by the local authorities. Additionally, these kindergartens and local villages were chosen based on their proximity to the health centre and because health teams routinely perform community health outreach activities, including child height measurement at these locations.’ (page 6, lines 120-124) I have left the text ‘Children were selected in random order to be measured’, (page 7, line 161) as it reflects the initial part of the measurement process. I have also left the sample size calculations as is (page 4, lines 88-91).

3) Regarding standardization test of the data collectors, please mention who was the gold standard measurer. Were there inter- and intra-rater reliability tests done during the training session? If yes, please add a sentence about the findings; 

Response: We did not have a gold standard measurer. Rather, we had a senior measurer with more experience who acted as the standard. We have revised the text on page 7, lines 146-153 under the ‘Training’ sub-heading, which now reads ‘The senior measurer was a study supervisor from the Lao TPHI team who is an experienced anthropometrist. Based on standardisation protocols, if a lead measurer showed a precision Technical Error of Measurement (TEM) of ≥0.4cm (our senior measurer had a TEM of 0.6cm), the criterion for trainee anthropometrists passing the standardization exercise was based on precision TEM within the limits of the Standardized Monitoring and Assessment for Relief and Transition (SMART) Manual 2.0 (22). Based on the standardization results, six of the twelve trained staff passed the protocol standards and became the measurers for data collection (precision TEMs between 0.6cm – 0.8cm).’ We used a combination of the WHO manual to guide our training and the SMART manual TEM range. We have added further detail on the TEMs assessed during the training. These TEMs were precision TEMs which showed intra-rater results. We did not assess inter-rater reliability during the training session as the precision (intra-rater) results were sufficient for training purposes. 

4) Please correct the name of the statistical method in line 207, it should be Bland-Altman; 

Response: Thank you for noticing this error. I have corrected it.

5) It might be better to split the section ‘Study design and ethics (line 90)’ into two distinct sections – ‘Study design and sample size calculation’ and ‘Ethical considerations’; 

Response: Thank you for this suggestion. We have revised both headings and split the sections as per your recommendation. 

6) Study design has not been mentioned in the ‘Study design’ section, please clearly mention the design of the study; 

Response: Thank you for this comment. We have included the study design more prominently in the manuscript. It is now written at the beginning of the ‘Study design and sample size calculation’ section as ‘We used a methods-comparison study design to assess the difference between the less-established digital device One Grows™ against the current standard, the wooden measurement board prescribed by UNICEF, currently the primary tool used around the world to measure height in low-resource contexts (17) .’ (Page 4, Lines 80-83). To create better flow within the document, you’ll notice that I have changed the order of the subsections in the Materials and Methods section.

7) In the ‘Study setting and participants’ section, please consider adding a few demographics of the Vientiane Province that may include geographical location, population, etc. 

Response: Thank you for this comment. I have included some brief details about the study location and added this sentence ‘We conducted this study in the district of Feuang in Vientiane Province, Lao People’s Democratic Republic (Lao PDR). Feuang is a district located 110 km by road from Vientiane capital city. According to the latest census from 2015, its population was just over 41,000 and 72% of the district was considered rural (19).’ (Page 5, lines 103-106) 

8) It would be good to provide information about the research group/institution/organization that conducted the study; 

Response: Thank you for this comment. I have included an additional sentence in the subsection entitled ‘Training’ on page 6, lines 128-132, it reads as follows: ‘The Lao TPHI is the primary public health research body in the country. They are a branch of the Ministry of Health and have been operational in a research and teaching capacity since 1999. When conducting research, the local level government encourages the use of local health workers to build their capacity research while being familiar with local contexts.’

9) The subheading “Training on use of One Grows™ device and Standardization Exercise for measurement” can be shortened. It can be simply “Training”; 

Response: Thank you. I have shortened this subsection title as suggested (page 6, line 126).

10) please consider using appropriate citations for the REDCap and StataSE 17 (line 147). 

Response: Thank you for your feedback. I have included the appropriate citations for both on page 8.

Further, we would like to request that you revise the text to fix the grammatical errors and improve the language usage and overall readability of the manuscript.

Response: Thank you for your attention to this detail. We have copyedited throughout the document.

1. Is the manuscript technically sound, and do the data support the conclusions?

The manuscript must describe a technically sound piece of scientific research with data that supports the conclusions. Experiments must have been conducted rigorously, with appropriate controls, replication, and sample sizes. The conclusions must be drawn appropriately based on the data presented.

Reviewer #1: Partly

Reviewer #2: Partly

________________________________________

2. Has the statistical analysis been performed appropriately and rigorously?

Reviewer #1: Yes

Reviewer #2: Yes

________________________________________

3. Have the authors made all data underlying the findings in their manuscript fully available?

The PLOS Data policy requires authors to make all data underlying the findings described in their manuscript fully available without restriction, with rare exception (please refer to the Data Availability Statement in the manuscript PDF file). The data should be provided as part of the manuscript or its supporting information, or deposited to a public repository. For example, in addition to summary statistics, the data points behind means, medians and variance measures should be available. If there are restrictions on publicly sharing data—e.g. participant privacy or use of data from a third party—those must be specified.

Reviewer #1: Yes

Reviewer #2: No

Response: The data collected is available on the Monash University repository website ‘Bridges’ as listed in the appropriate section of the online submission You can find it here: https://doi.org/10.26180/22678663.v1

________________________________________

4. Is the manuscript presented in an intelligible fashion and written in standard English?

PLOS ONE does not copyedit accepted manuscripts, so the language in submitted articles must be clear, correct, and unambiguous. Any typographical or grammatical errors should be corrected at revision, so please note any specific errors here.

Reviewer #1: Yes

Reviewer #2: Yes

________________________________________

5. Review Comments to the Author

Please use the space provided to explain your answers to the questions above. You may also include additional comments for the author, including concerns about dual publication, research ethics, or publication ethics. (Please upload your review as an attachment if it exceeds 20,000 characters)

Reviewer #1: This paper compares two methods, a wooden height meter board and an ultrasound device, by measuring height in children aged 2-5 years in Lao PDR.

The article describes the results, assessing TEM for precision and accuracy and comparing the results with the WHO MGRS and SMART manuals.

The focus of the paper is well phrased.

I recommend that this paper be accepted after minor revision.

Minor comments:

1. It is mentioned that 12 health workers were trained in the measurement of height using ultrasound equipment (line 22). On the other hand, it is noted that only 6 of these workers passed the protocol standards and served as measurers in the study (line 125). Therefore, the above statement may be misleading.

Response: Thank you for this comment. We have revised this section of text which now reads ‘Since each team required a measurer and a scribe (someone to write down the result), we required six teams in total. Based on the standardization results, six of the twelve trained staff passed the protocol standards and became the measurers for data collection. The remaining six staff became measurement recorders.’ (page 7, lines 153-155)

2. Ethical aspects need to be described in detail. It is stated that it was convenience sampling (line 88) and that consent was obtained from parents or caregivers (line 89), but the specifics are not clear from these descriptions. Although this study involves recruiting subjects in kindergartens and villages in Lao PDR, it is unclear whether sufficient consideration was given to the subjects regarding their permission to participate. The fact that the health team routinely conducts outreach activities may have influenced subjects' willingness to participate. Therefore, the measures to address this should also be described.

Response: Thank you for this feedback. Based on comments from the editor, we have removed the text about convenience sampling and rephrased this in the ‘Study setting and participants’ section (page 5-6) to be clearer about our methodology. It now reads (page 6, lines 120-124) ‘Participating children were identified and recruited from two kindergartens and three villages near the local health centre, deemed suitable study sites by the local authorities. Additionally, these kindergartens and local villages were chosen based on their proximity to the health centre and because health teams routinely perform community health outreach activities, including child height measurement at these locations’. 

Regarding the Ethics subsection, we have included more detail on this as suggested. The text now reads (on page 5, lines 96-101) ‘Prior to the study taking place, permission was sought by the local study team to conduct the study from the relevant district authorities and each study site. In the weeks leading up to the data collection taking place, authorities from the study site informed the parent and caregivers of the child participants of our study. On the morning of the data collection, written informed consent was received from each child’s parent/caregiver at the study sites before measurements were taken.’ The study protocol was approved by the Human Research Ethics committees in Australia and in Lao PDR. It was advised by local authorities when we developed our study protocol that selecting sites familiar to the health outreach teams were an advantage to the families and their children. Given the assessment of height is a very low risk activity, having familiar faces conduct the measurements with a novel device was deemed safer for the children. 

3. The text does not describe any differences among assessors. It is anticipated that this data would provide a better understanding of the comparison between the two height measurement methods.

Response: Thank you for this comment. We have included additional text in our ‘Results’ section (page 13) under a new sub-heading ‘Team results’ (lines 274-279). This was not originally included as the study methodology was not designed to assess between-team variation, but to compare the two devices as is. Given this, there was variation in the number of children (between 19 to 75) that each assessor measured. As such, the results among individual assessors do not have statistical power to provide a meaningful interpretation or comparison to each other. 

In lieu of this, we have included a range of the precision and accuracy results which indicated that both tools were reliable and within acceptable limits. As we were assessing the teams' results, we corrected a small data analysis error for measurement board results among the boys. This meant a very minor change in the results (Table 5 (last two columns), page 13, lines 268-269) for the ‘Precision TEM Measurement Board (Males and All)’ and ‘Accuracy TEM (Males and All)’. The results for the Precision TEM of the ultrasound device and girls remain the same. These minor edits had no effect on the conclusion. 

4. The use of ultrasound device in resource-limited settings is described (line 272). However, durability as well as convenience are important factors in making better choices for clinical use in these environments. Is this point included in the discussion?

Response: Thank you for this comment. We mention throughout the manuscript that the ultrasound device is a handheld, portable device which is less bulky than the measurement board. We have also added a photograph of the device (Fig. 1). We have included some additional text in the ‘Discussion’ section (page 15, lines 317-321) which now reads ‘The One Grows™ device meets both UNICEF TPP essential requirements. It also meets several optional requirements including an associated mobile application (app) whereby the measurement taken by the device is automatically entered via Bluetooth technology, and requirements for operational conditions, portability, maintenance, physical characteristics, and affordability’.

Reviewer #2: There are several points in the manuscript to be made clearer.

1. Although the authors describe "Aspects such as correct head and feet positioning (the same when using the measurement board) were key aspects" in lines 116-117, the height measurement by One Grows may be not familiar to readers. Please, add the further information on the logic of One Grows.

Response: Thank you for this comment. I have included further information on the technology of the One Grows device as well as an additional figure (Fig. 1), a photograph of the device to assist readers. Please see page 5, lines 109-115 where the logic of One Grows is now explained as ‘The One Grows™ is not currently patented (Fig. 1) and measures height by sending ultrasound beams from the head of the device to a hard surface. To measure standing height, the device is placed perpendicularly against a wall, facing down on top of the child’s head. The child is then asked to step away from their position and the measurer presses the button on the device to allow it to take the measurement. The measurement is converted into centimetres by the device by calculating the time it takes for the beam to bounce to/from the hard surface.’

2. In line 126, the authors describe "six measurement teams". Were there differences in precision and accuracy among the teams? Please, mention them in Results.

Response: Thank you for this comment. Please see response above, presented to Reviewer #1, response #3.

3. In this study, one team measured height with the two methods alternatively but consecutively. The staff could memorize the measured height, resulting in the reduced difference among six measurements. Please, discuss the effect.

Response: Thank you for this feedback. We recognised this limitation and is included in our discussion section as a limitation on page 17, lines 370-373, which reads ‘Our measurement process of alternating devices for the same child could contribute to measurers being biased by previous measurements. To reduce this bias, in future studies, it may be worth considering the measurement of an entire group of children once with both devices, then re-measuring the same group of children another time over.’

4. The authors stated that the subjects were 222 children in the text, but Table 5 shows 210 females, 134 males, and 444 in total.

Response: You are correct. This is because these are the calculations of two measurements. Given the sample size n=222, the two measurement per child equates to n=444 measurements analysed. However, we can see that this may be confusing so have indicated that n= number of children, not number of measurements. We have clarified this in the table of results on Table 5 page 13.

5. In line 261, the authors stated "The ultrasound device produced a systematic bias of 0.1cm", as well as in line 30, but there is no description on it in Results.

Response: Thank you for this comment. The bias of 0.1cm is the mean difference of measurements as represented in Table 4 on page 12, line 241.

Technical errors are as below.

1. In line 32, "The ultrasound and board TEMs for precision were 0.157cm and 0.113 respectively. ", "cm" may be needed after "0.113". 

Response: Corrected.

2. There is no Table 2 after line 201. 

Response: Corrected.

3. It is better to replace "Table 5: Precision and bias of ultrasound and measurement board" with "Table

5: Precision and accuracy of ultrasound and measurement board". 

Response: Edited as per suggestion.

This is a copy and paste of the document uploaded labeled 'Response to Reviewers'.

Attachment Submitted filename: Response to reviewers.docx Click here for additional data file.

# Editor report: Decision Letter 1

**DOI:** 10.1371/journal.pone.0289514.r003

**Authors:** Sayedur Rahman

27 Oct 2023

Comparing the accuracy of an ultrasound height measurement device with a wooden measurement board among children aged 2-5 years in rural Lao People’s Democratic Republic: a methods-comparison study.

PONE-D-23-15762R1

Dear Dr. Huang,

We’re pleased to inform you that your manuscript has been judged scientifically suitable for publication and will be formally accepted for publication once it meets all outstanding technical requirements.

Within one week, you’ll receive an e-mail detailing the required amendments. When these have been addressed, you’ll receive a formal acceptance letter and your manuscript will be scheduled for publication.

An invoice for payment will follow shortly after the formal acceptance. To ensure an efficient process, please log into Editorial Manager at http://www.editorialmanager.com/pone/, click the 'Update My Information' link at the top of the page, and double check that your user information is up-to-date. If you have any billing related questions, please contact our Author Billing department directly at authorbilling@plos.org.

If your institution or institutions have a press office, please notify them about your upcoming paper to help maximize its impact. If they’ll be preparing press materials, please inform our press team as soon as possible -- no later than 48 hours after receiving the formal acceptance. Your manuscript will remain under strict press embargo until 2 pm Eastern Time on the date of publication. For more information, please contact onepress@plos.org.

Kind regards,

Sayedur Rahman, MBBS, MMSc

Academic Editor

PLOS ONE

Additional Editor Comments (optional):

Reviewers' comments:

# Editor report: Acceptance letter

**DOI:** 10.1371/journal.pone.0289514.r004

**Authors:** Sayedur Rahman

9 Nov 2023

PONE-D-23-15762R1 

Comparing the accuracy of an ultrasound height measurement device with a wooden measurement board among children aged 2-5 years in rural Lao People’s Democratic Republic: a methods-comparison study. 

Dear Dr. Huang:

I'm pleased to inform you that your manuscript has been deemed suitable for publication in PLOS ONE. Congratulations! Your manuscript is now with our production department. 

If your institution or institutions have a press office, please let them know about your upcoming paper now to help maximize its impact. If they'll be preparing press materials, please inform our press team within the next 48 hours. Your manuscript will remain under strict press embargo until 2 pm Eastern Time on the date of publication. For more information please contact onepress@plos.org.

If we can help with anything else, please email us at plosone@plos.org. 

Thank you for submitting your work to PLOS ONE and supporting open access. 

Kind regards, 

PLOS ONE Editorial Office Staff

on behalf of

Dr. Sayedur Rahman 

Academic Editor

PLOS ONE
